# Postpartum Thyroiditis Mimicking Central Hypothyroidism: The Perfect Thyming

**DOI:** 10.7759/cureus.42630

**Published:** 2023-07-28

**Authors:** James R Kang, Rachel Cox, Joseph Kluesner

**Affiliations:** 1 Internal Medicine, Wright State University Boonshoft School of Medicine, Dayton, USA; 2 Internal Medicine, Wright-Patterson Air Force Base, Dayton, USA; 3 Internal Medicine, Wright State University, Dayton, USA; 4 Endocrinology, Wright-Patterson Air Force Base, Dayton, USA

**Keywords:** hypothyroidism, primary hypothyroidism, central hypothyroidism, auto-immune thyroiditis, postpartum thyroiditis, postpartum complication

## Abstract

Hypothyroidism can be seen in postpartum women as a result of central or primary hypothyroidism. Postpartum thyroiditis is a primary hypothyroid condition in which there is likely autoimmune dysfunction leading to damage to the thyroid gland. Patients with postpartum thyroiditis often present with little to no symptoms, and the key to establishing this diagnosis is a comprehensive endocrine workup. We report the case of a 24-year-old postpartum female patient who was diagnosed with postpartum thyroiditis after initial evaluation demonstrated findings concerning central hypothyroidism.

## Introduction

Hypothyroidism presenting in the postpartum period can be due to central hypothyroidism or primary hypothyroidism. Examples of central hypothyroidism seen postpartum include Sheehan’s syndrome, which occurs as a result of hypoperfusion of the pituitary secondary to hemorrhagic hypovolemic shock, and lymphocytic hypophysitis, which is a rare autoimmune condition that causes lymphocytic invasion of the pituitary [1,2]. The serum levels of thyroid-stimulating hormone (TSH) are typically inappropriately low or normal with low levels of free thyroxine (FT4) or free triiodothyronine (FT3). Because there is pituitary dysfunction in central hypothyroidism, other pituitary hormone lines can be affected in these conditions. One of the important pituitary hormones that should be investigated in these patients is the adrenocorticotropic hormone (ACTH). This is because levothyroxine replacement therapy in the setting of untreated ACTH deficiency can lead to a life-threatening adrenal crisis by enhancing cortisol clearance [3].

In contrast, primary hypothyroidism presenting in the postpartum period can be due to postpartum thyroiditis (PPT), which is caused by autoimmune-mediated injury to the thyroid gland itself. Patients can present with transient hyperthyroidism, transient hypothyroidism, or a triphasic pattern manifesting as transient hyperthyroidism followed by hypothyroidism and eventual recovery [4,5]. Two distinguishing laboratory findings that can be seen in PPT are elevated serum levels of thyroid peroxidase antibody (TPO Ab) and thyroglobulin antibody (TGAb). The serum levels of TSH are typically high with normal or low serum FT4 levels during the hypothyroid phase.

We present the case of a 24-year-old postpartum female who presented with multiple symptoms concerning hypothyroidism. Initial thyroid function test (TFT) was suggestive of a central etiology, however further workup demonstrated the cause to be PPT. The following case report demonstrates the importance of a thorough investigation to help delineate the potential causes of postpartum thyroid dysfunction.

## Case presentation

A 24-year-old female patient with newly diagnosed hypothyroidism presented to the endocrinology clinic for evaluation and assistance in management. She had a miscarriage four months prior in the setting of placental abruption, requiring an emergent C-section at 24 weeks gestation. The patient otherwise had no significant medical comorbidities. Since the miscarriage, the patient had experienced many symptoms including weakness, fatigue, cold intolerance, hair loss, insomnia, depression, and anxiety. In addition to undergoing psychiatric and rheumatologic evaluation, the patient’s TFTs were checked one month prior to her presentation to the clinic. Resultant TSH and FT4 levels were 0.171 mIU/L and 0.61 ng/dL (lab reference ranges for TSH 0.4-4.0 mIU/L and for FT4 0.7-1.9 ng/dL), respectively, and the patient was started on levothyroxine 100 mcg daily by her primary care provider. 

At her initial encounter in the endocrinology clinic just two weeks after referral, the patient’s presenting TFT pattern was noted to be concerning for central hypothyroidism in the setting of the recent history of significant blood loss due to placental abruption requiring emergent C-section. A full biochemical workup of other pituitary cell lines returned normal and repeat TSH and FT4 levels one month after the initial assessment were 132 mIU/L and 0.14 ng/dL, followed by 80.9 mIU/L and 0.45 ng/dL one week later (Table 1). As the patient’s TSH was significantly elevated during her evaluation in the setting of classic hypothyroidism symptoms, she was continued on levothyroxine with a plan to titrate levothyroxine to achieve normal TSH. However, the patient was lost to follow-up.

**Table 1:** Additional laboratory tests demonstrated elevated levels of TPO Ab and TGAb with intact pituitary hormone lines in the setting of TFTs that were consistent with primary hypothyroidism. *250 mcg of cosyntropin administered intravenously or intramuscularly followed by serum cortisol level check at 30 minutes and at 60 minutes TPO Ab: Thyroid peroxidase antibody, TGAb: Thyroglobulin antibody, IGF-1: Insulin-like growth factor 1, ACTH: Adrenocorticotropic hormone, FSH: Follicle-stimulating hormone, LH: Luteinizing hormone, DHEA-S: Dehydroepiandosterone sulfate

Laboratory test	Serum level	Lab reference range
TPO Ab	>450	<34 IU/mL
TGAb	379	0-10 IU/mL
Cortisol after cosyntropin stimulation*	28.7 at 30 minutes; 23 at 60 minutes	>18 mcg/dL
IGF-1	164	171-459 ng/mL
ACTH	12.5	10-60 pg/mL
FSH	6.3	<7 mIU/mL
LH	15.5	5-25 mIU/mL
Prolactin	15	<25 ng/mL
Morning cortisol	5.81	5-25 mcg/dL
DHEA-S	104	65-380 mcg/dL

## Discussion

In postpartum patients with symptoms of hypothyroidism, TFTs should be promptly obtained. Moreover, the data should be interpreted cautiously as primary hypothyroidism could be easily confused with central hypothyroidism. The overall mean prevalence of PPT is estimated at 7.2%, but this statistic varies geographically [6]. Our patient’s initial TFTs were suggestive of a central etiology with low TSH and FT4, but extensive biochemical workup revealed that her pituitary cell lines were normal with repeat TFTs later showing elevated TSH with low FT4, a pattern consistent with primary hypothyroidism secondary to PPT. Her initial TFTs most likely reflected a transition period between a hyperthyroid and hypothyroid phase during PTT. 

PPT refers to thyroid dysfunction, other than Graves disease, that develops during the initial year after childbirth in women who had normal thyroid function before pregnancy [7]. It is considered a variant of Hashimoto's thyroiditis and can present in different ways. PPT can present in a triphasic pattern in which patients present with a hyperthyroid phase occurring one to six months postpartum and lasting roughly 16 weeks. This follows by a hypothyroid phase also lasting roughly 16 weeks and finally returns to an euthyroid state [7,8]. However, around 10%-20% of patients do not recover and can have persistent hypothyroidism [4]. TFTs obtained in the transition of these phases can be misleading. In the hyperthyroid phase, TSH declines and can be undetectable due to suppression from elevated thyroid hormone levels. During this transient phase, treatment is typically targeted for symptoms of hyperthyroidism in the form of beta-blockade. After the resolution of inflammation and exhaustion of FT4 stores, FT4 levels decline and can be low. Recovery of TSH release by the pituitary can be delayed which can lead to a brief period in which levels of both TSH and FT4 are low, mimicking the TFT pattern of central hypothyroidism as demonstrated in our patient. Within weeks, TSH levels recover and become elevated, thus matching the typical pattern of primary hypothyroidism. If hypothyroid symptoms are present during this phase, the patient can be treated with levothyroxine. A typical treatment with thyroid hormone replacement lasts for six to 12 months with consideration of a gradual taper depending on the patient’s response to treatment and recovery of thyroid function. It should be noted that tapering of levothyroxine should be avoided in patients who are pregnant or actively attempting pregnancy.

In our presented case, repeating the patient’s TFTs in a timely manner and assessing other pituitary hormone lines in the evaluation led to the appropriate diagnosis and long-term treatment plan. Of note, patients with a history of PPT are at risk for recurrence following future pregnancies, so all patients should be counseled and TFTs should be monitored with each pregnancy. Lastly, patients with PPT can develop hypothyroidism even with the initial recovery of their thyroid function and should likely have their TSH levels checked annually moving forward [4].

## Conclusions

In postpartum patients presenting with symptomatic hypothyroidism without known autoimmune disease or history of PPT, it is essential to differentiate between a primary and central etiology of hypothyroidism. TFTs in patients with PPT can be misleading due to PPT’s triphasic nature and it is helpful to broaden the workup to include other pituitary hormone lines and timely repeating of TFTs, as evidenced in this case. Our patient case highlights the importance of performing a thorough workup while evaluating and treating postpartum thyroid dysfunction.
